# The Role of Large-Format Histopathology in Assessing Subgross Morphological Prognostic Parameters: A Single Institution Report of 1000 Consecutive Breast Cancer Cases

**DOI:** 10.1155/2012/395415

**Published:** 2012-10-21

**Authors:** Tibor Tot

**Affiliations:** Department of Pathology and Clinical Cytology, Central Hospital Falun, 791 82, Falun, Sweden

## Abstract

Breast cancer subgross morphological parameters (disease extent, lesion distribution, and tumor size) provide significant prognostic information and guide therapeutic decisions. Modern multimodality radiological imaging can determine these parameters with increasing accuracy in most patients. Large-format histopathology preserves the spatial relationship of the tumor components and their relationship to the resection margins and has clear advantages over traditional routine pathology techniques. We report a series of 1000 consecutive breast cancer cases worked up with large-format histology with detailed radiological-pathological correlation. We confirmed that breast carcinomas often exhibit complex subgross morphology in both early and advanced stages. Half of the cases were extensive tumors and occupied a tissue space ≥40 mm in its largest dimension. Because both in situ and invasive tumor components may exhibit unifocal, multifocal, and diffuse lesion distribution, 17 different breast cancer growth patterns can be observed. Combining in situ and invasive tumor components, most cases fall into three aggregate growth patterns: unifocal (36%), multifocal (35%), and diffuse (28%). Large-format histology categories of tumor size and disease extent were concordant with radiological measurements in approximately 80% of the cases. Noncalcified, low-grade in situ foci, and invasive tumor foci <5 mm were the most frequent causes of discrepant findings.

## 1. Introduction

Breast cancer is a heterogeneous group of diseases which deviate from each other in natural history, morphology, molecular phenotype, clinical and radiological manifestations, and prognosis. Prognostic parameters are essential for predicting the outcome and response to therapy in individual cases. The long list of more or less powerful prognostic parameters that includes patient age, mode of detection, tumor size, histologic grade, lymph node status, and presence or absence of distant metastases was recently widened with molecular tumor phenotypes assessed with either genetic tests or immunohistochemistry. Since the number of therapeutic options is rather limited, the parameters for which assessment is routinely required for therapeutic decisions are also few. Whereas hormone receptor status, HER-2 status, and proliferative activity are the major determinants of oncological therapy, proper characterization of the subgross morphology of breast carcinoma is essential for planning appropriate surgery and radiation therapy [1–4]. The prognostic significance of subgross parameters is also observed [1, 4, 5].

For correct subgross characterization of a case, the following parameters should be assessed: tumor size (defined as the largest diameter of the largest invasive focus), lesion distribution (unifocal, multifocal, or diffuse distribution of the invasive and in situ tumor components), disease extent (corresponding to the tissue volume containing all the malignant structures within the breast), intratumoral or intertumoral heterogeneity, and the position of the tumor within the breast [5]. These parameters can be assessed with radiological and histopathological methods, the most efficient being a combination of these methods in the form of detailed and systematic radiological-pathological correlation [5–10].

An applied histopathology method substantially influences the success rates of documenting and assessing this subgross morphological parameters and correlating them to radiological findings. The traditional small block sampling method is based on taking 1-2 cm sized samples from breast specimens, often under the control of only the pathologist's naked eye and sometimes using radiological guidance. This way, the specimen is fragmented and the interrelationship of the different tumor components, which are not represented in the same block, is destroyed. Taking large numbers of small blocks, sequential numbering of the blocks, and marking the sample placement on a macrophotograph of the specimen or in the specimen radiograph represent attempts to compensate for the obvious limitations of the sampling method. At the same time, these attempts are proof that such compensation is necessary. Large-format histopathology is based on embedding and processing contiguous tissue slices representing the entire cross section of a segmentectomy specimen, preserving the interrelationships of the components of the tumor, and documenting them together in one plane. This advantage makes this method the best approach in correctly assessing the subgross morphological parameters, which also facilitates the detailed radiological-pathological correlation [5, 6, 8–10]. This technique has been successfully adapted to the needs of busy routine laboratories and the procedure has been repeatedly described in detail [5, 6, 11–13]. The advantages of this method have also been observed in a recent cost-benefit analysis [14].

## 2. Documenting the Extent of the Disease

Defined as the tumor volume containing all the actual malignant structures within the breast, the extent of disease is the most important subgross parameter influencing the feasibility of breast-conserving surgery in an actual case [15]. This is the volume of breast tissue the surgeon aims to remove within certain margins in order to prevent local recurrences. Disease extent that is ≥40 mm in the greatest dimension is associated with an approximately three-fold risk of ipsilateral local recurrence after breast conserving surgery and irradiation compared with those cases with disease extent limited to a volume of <40 mm [4, 16]. In addition, patients with extensive disease (≥40 mm in the largest dimension) have significantly decreased long-term disease-specific survival compared with those with tumors of limited extent [17]. All this underlines the importance of correctly assessing the subgross morphological prognostic parameter.

In everyday routine, the pathologist should begin the analysis of a case by recapitulating the radiological findings, including the radiological disease extent. The next step should be comparing the uncut specimen with the whole specimen radiograph and keeping the *in vivo* orientation of the specimen by inking it at its margins [11]. 

Breast cancer is a lobar disease most often involving parts of a single sick lobe [18, 19]. The lobe is a pyramid-like structure, with the lactipherous duct opening in the nipple, branching in the direction of the pectoralis muscle, and ending up in a large number of terminal units. In order to demonstrate the largest cross-section of the involved lobe, the segmentectomy specimen has to be sliced into 3-4 mm slices parallel to the pectoralis fascia, but not perpendicular to it. The perpendicular slicing method leads to a substantial underestimate of the extent of the disease in the vast majority of ductal carcinoma in situ cases [20, 21]. 

The space the malignant structures occupy in the breast rarely shows the regular shape of a geometric body; it is almost always irregular. This means that the borders of this space are different at different levels of the specimen and in different projections. Consequently, the area representing the cross section of this tissue space in the tissue slices of the specimen also varies. For correct visualization of the real disease extent, the slice with the largest disease area should be chosen (based on the specimen radiograph and macroscopy), embedded, and processed; but additional levels should also be embedded because some components of the disease may not be visible on imaging and macroscopy [11]. 

The microscopic analysis should begin with determining the disease extent. Approaching from the periphery of the section, the pathologist should mark the most peripheral malignant structures (in situ or invasive) and repeat the process from all directions. The result will be a marked area representing a cross section through the diseased tissue. Summarizing the findings in adjacent tissue slices and/or tissue slices taken at different levels of the specimen is often necessary. Correlating the radiological and histological findings is essential [11]. The realistic aim of the disease extent assessment is an appropriate categorization of the tumor as of extensive (occupying a space ≥40 mm) or limited (occupying a space <40 mm) extent rather than achieving millimetric concordance of the radiological and the histological extent.

In a consecutive series of 1000 newly diagnosed breast cancers in our material (Central Hospital Falun, Sweden, period Dec 2007 to Jun 2012), 495 cases were extensive and occupied a tissue volume of ≥40 mm in the greatest dimension and 505 were nonextensive occupying smaller tissue volumes. Purely in situ carcinomas together with microinvasive (<1 mm, 4 cases) tumors comprised 14% (144/1000) of the series, and half of the cases were extensive (48%, 69/144) and half were nonextensive (52%, 75/144). Early invasive carcinomas (1–14 mm) comprised 35% (349/1000) of the series; 42% (146/349) were extensive, and 58% (203/349) were nonextensive. In more advanced cancers (≥15 mm in size, 50% of the series, 500/1000), 55% (273/500) of the cases were extensive (Table 1).

## 3. Assessing Lesion Distribution

After the extent of the disease is characterized as described in Table 1, the pathologist should judge whether the lesions within the tissue area are individual (well demarcated and separate from each other) or confluent (inseparable). This judgment is easier if the invasive tumor component(s) and the in situ component(s) are assessed separately. A simple practice of encircling the separable invasive foci with one color and the separable in situ foci with another color is helpful. While characterization of the foci requires microscopic control, the judgment of lesion distribution after the individual lesions are marked must be carried out using a naked eye examination of the large-format histology sections, without using a microscope.

On the preoperative tumor board, the pathologist should register the radiological lesion distribution and plan the dissection of the specimen on the basis of this information. Radiologically unifocal lesions are usually properly represented in one or two large-format histology sections, provided that one of these contains the tumor at its largest cross-section [11]. In radiologically multifocal cases, several slices should be embedded to visualize as many tumor foci as possible. In radiologically diffuse cases, the most important task is to visualize the correct extent of the disease and, if the diffuse component is in situ, to catch the radiologically or macroscopically evident invasive component(s). 

**Figure 1:**
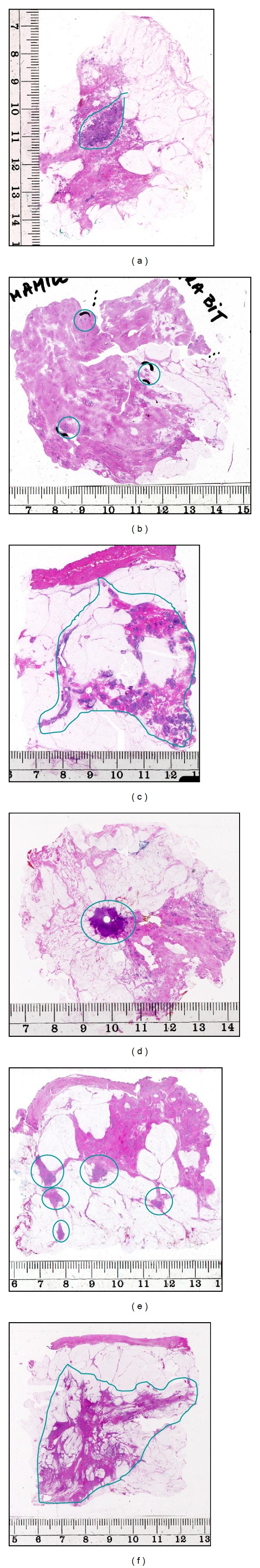
The basic breast cancer growth patterns. (a) Unifocal in situ carcinoma: the tumor involves neighboring terminal ductal-lobular units. (b) Multifocal in situ carcinoma: the tumor involves distant terminal ductal-lobular units. (c) Diffuse in situ carcinoma: the tumor involves large ducts and many terminal ductal-lobular units. (d) Unifocal invasive carcinoma: a single well-delineated invasive focus. (e) Multifocal invasive carcinoma: several well-delineated invasive foci in the same specimen. (f) Diffuse invasive carcinoma: poorly delineated, spider's web-like structure. All the malignant lesions are encircled.

**Figure 2:**
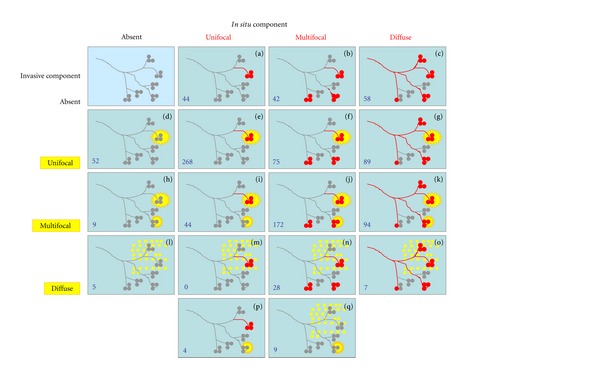
Schematic illustration of the possible combined growth patterns in breast carcinomas. (a) Unifocal in situ component, no invasive component. (b) Multifocal in situ component, no invasive component. (c) Diffuse in situ component, no invasive component. (d) Unifocal invasive component, no in situ component. (e) Unifocal invasive component, unifocal in situ component within the area of the invasive focus, and unifocal combined pattern. (f) Unifocal invasive component, multifocal in situ component, and multifocal combined pattern. (g) Unifocal invasive component, diffuse in situ component, and diffuse combined pattern. (h) Multifocal invasive component, no in situ component. (i) Multifocal invasive component, unifocal in situ component in one of the invasive foci, and multifocal combined pattern. (j) Multifocal invasive component, multifocal in situ component, and multifocal combined pattern. (k) Multifocal invasive component, diffuse in situ component, and diffuse combined pattern. (l) Diffuse invasive component, no in situ component. (m) Diffuse invasive component, unifocal in situ component, and diffuse combined pattern. (n) Diffuse invasive component, multifocal in situ component, and diffuse combined pattern. (o) Diffuse invasive component, diffuse in situ component, and diffuse combined pattern. (p) Unifocal invasive component, unifocal in situ component outside the invasive focus, and multifocal combined pattern. (q) Drawing illustrating one of the possible mixed patterns with both diffusely growing and well-delineated invasive foci, with a diffuse combined pattern. The upper right image illustrates the sick lobe. Numbers in the lower left corner of the drawings indicate the number of cases in the series of 1000 consecutive breast carcinomas belonging to that category.

Our previously published system is the only one that takes into account both the invasive and in situ components of the tumor and defines their distribution both individually and in combination [22]. In addition, our system recognizes the diffuse distribution of both the in situ and invasive tumor components, in contrast with other systems described in publications on breast cancer multifocality [1, 23]. In our system, invasive lesions are considered “unifocal” if only one invasive focus can be observed that is well delineated and may or may not contain an in situ component. “Multifocal” invasive lesions are characterized by the presence of multiple, well-delineated, invasive tumor foci separated from each other by uninvolved breast tissue containing normal tissue, benign lesions, or in situ carcinoma, regardless of the distance between the invasive foci. Tumors dispersed over a large area with no distinct tumor mass, for example, like a spider's web, are classified as “diffuse.” In situ carcinomas are regarded as “unifocal” if they appear to involve a single terminal ductal lobular unit (TDLU) or several neighboring TDLUs. In situ carcinomas are designated “multifocal” if they involve several distant TDLUs with uninvolved breast ducts and TDLUs in between and as “diffuse” if they mainly involve the larger ducts [22]. The distribution of the invasive and in situ components is then combined so that a diffuse distribution of either the in situ or the invasive component qualifies the lesion to be categorized as “diffuse.” Multifocality of the invasive and/or in situ component indicates a “multifocal” designation. Typical cases with unifocal, multifocal, and diffuse in situ and invasive breast carcinomas are illustrated in Figure 1. As shown in Figure 2, there are 17 different combined distribution patterns in breast carcinomas (unifocal, multifocal, diffuse, or missing in situ component combined with the same invasive categories, plus a mixed category). Although the combined pattern of lesion distribution in breast carcinomas is not always easy to assess, and higher levels of interobserver reproducibility may require substantial experience [24], the combinations reduce the 17 different pattern possibilities to 3 aggregate patterns.

Multifocality is often described in the literature as the presence of satellite tumors around and in the vicinity of a dominant mass [25]. Although this situation is common, the concept is erroneous because there are cases with multiple tumor foci of approximately the same size, without the presence of a dominant mass. These foci may be dispersed over a large area without the tendency to concentrate around one foci. With regard to their evolution, two different types of multifocal invasive cancer may exist: one with multiple individual invasive foci, which develops from in situ lesions at different parts of the same lobe simultaneously or with a time difference, and one in which the individual foci represent “in transit” metastases [26] of a primary focus and are not related to an in situ component.

**Figure 3:**
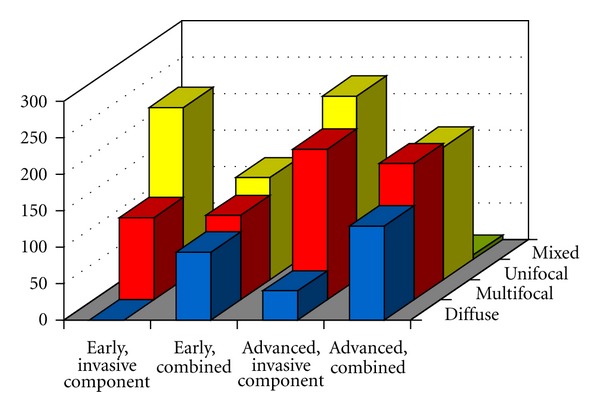
Distribution of the invasive component and combined (in situ plus invasive) lesion distribution in 855 consecutive invasive breast carcinoma cases documented in large-format histology slides. Falun, Dec 2007 to Jun 2012.

The cases in our series of 1000 breast carcinomas showed the following combined lesion distribution: unifocal in 36% (366/1000), multifocal in 35% (347/1000), and diffuse in 28% (280/1000), as shown in Table 1. In addition, there were 7 cases with mixed or undetermined lesion distribution. In situ carcinomas, including 4 cases of microinvasive tumors, were unifocal in 31% (44/144), multifocal in 28% (42/144), and diffuse in 41% (58/144) of cases. The majority (68%, 236/349) of the early invasive cancers (<15 mm in size) had a unifocal invasive component, but when the combined morphology of the in situ and invasive components was taken into account, the majority (60%, 209/349) were in fact multifocal or diffuse. Approximately one-third of more advanced (≥15 mm in size) breast carcinomas (36%, 182/500) had unifocal combined (in situ plus invasive) morphology, one-third (38%, 189/500) had multifocal, and the remainder (25%, 129/500) had diffuse-combined lesion distributions, mainly because the diffuse in situ component (Figure 3). Diffuse invasive cancers were rare. These data are in full agreement with our previously published results [22, 27–30] and are similar to the results of other studies based on an analysis of large-format histology slides [31, 32].

Testing the prognostic significance of the lesion distribution defined above has resulted in clear separation of the unifocal, multifocal, and diffuse tumors with regards to the invasive component, the in situ component, and the combined distribution. Patients with multifocal or diffuse invasive carcinomas have a more than double risk of lymph node metastasis compared with unifocal tumors [22, 28–30, 33, 34], and the differences are related to macrometastatic disease [35]. Differences in disease-specific survival are also evident; patients with diffuse invasive or diffuse combined tumor growth patterns have a worse outcome, those with multifocal disease an intermediate outcome, and those with unifocal tumors have the best long-term outcome [17]. A worse survival of patients with multifocal tumors was also observed in both early [36] and recent studies [23, 37]. 

By stereomicroscopic examination of large-format thick histological sections, Foschini at al. demonstrated that the distance between the individual foci of some low-grade in situ carcinomas is more than 20 mm indicating the possibility that these foci are located within different lobes [32]. Although some breast lobes are large and widespread, synchronous or asynchronous development of a carcinoma in different lobes of the same breast is a real possibility. These multilobar/multicentric cases are regularly associated with multiplicity of tumor foci and with large disease extent. In practice, the above described rules of assessing the lesion distribution and disease extent are also applicable in the multilobar cases.

## 4. Documenting Tumor Size

Tumor size is defined as the largest diameter of the largest invasive tumor focus [25] and represents one of the most powerful prognostic parameters, a constituent of the TNM staging system. Many studies document its prognostic significance, and the larger the tumor, the purer the prognosis. This represents the basis for the success of mammography screening by finding tumors at an earlier stage of their natural history when they are still small, improving the overall prognosis of breast cancer patients in the screened population [38]. In addition to purely in situ carcinomas, microinvasive cancers, which have invasive foci <1 mm, and invasive carcinomas <15 mm belong to the category of early breast carcinomas [39, 40]. Patients with these tumors have an excellent, over 90%, 10-year disease-specific survival [40] and, provided that they are detected by mammography screening, the overall survival of these patients does not differ from the survival of age-matched women in the general population [41]. Forty nine percent of cases in our material were classified in this category: 14% (144/100) were in situ and in situ with microinvasive cancers, and 35% (349/1000) were invasive carcinomas of <15 mm. More advanced cancers have an invasive component measuring ≥15 mm. Patients with these tumors have less favorable survival outcomes compared with early breast cancer cases [39, 40]. The proportion of cases in our material classified in this category was 51% (500/1000 unifocal, multifocal, or diffuse cases plus 7 cases with mixed growth patterns) (Table 1).

**Figure 4:**
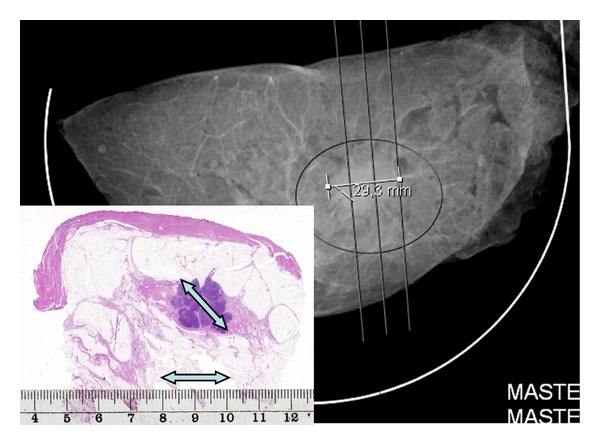
Mastectomy specimen-large-format histopathology correlation: unifocal invasive cancer. The plane of slicing the mastectomy specimen was erroneously chosen, which resulted in a discrepantly smaller tumor size in the histology slide compared with the mammographic size of the specimen. The specimen mammography image is courtesy of Dr. Mats Ingvarsson.

Determining tumor size is a complex task. The pathologist should register the radiologically measured tumor size on the preoperative tumor board. Breast cancers are often irregular in shape, such that the largest diameter of their nongeometric body varies in different projections. During the dissection, the pathologist should attempt to slice the specimen so that the cross section with the largest diameter of the tumor can be visualized (see Figure 4) and to document it in its entirety in a large section, without fragmenting the tumor. Embedding slices at different levels of the specimen and summarizing the findings in different slides are as important as in determining the extent of the disease [11].

Radiological methods, especially modern ultrasound and magnetic resonance imaging, provide an accurate measure of the size of the tumor in several projections. The main shortcoming of these otherwise very accurate measurement methods is that they do not always distinguish in situ and invasive parts of the same tumor; because of this, the histologically verified tumor size may deviate from the radiological one. Obvious additional discrepancies between the radiological and histological tumor size may be the result of preoperative neoadjuvant therapy, but can also be caused by an erroneous choice of the embedded slice during the dissection or result from a failure in the radiological-pathological correlation.

There is no international consensus about measuring tumor size; for example, as a size restricted to measuring the tumor body or including the invasive extensions (spiculations). Because the spiculations may be long but are usually thin, they contain invasive cancer representing only a minor part of the tumor burden. Including such extensions when measuring tumor size may lead to an overestimate of the tumor burden. The aim of the tumor size measurement should be to categorize the case as early (<15 mm in size) or more advanced, rather than to expect a millimetric concordance of radiological and histopathological findings. 

## 5. Radiological-Pathological Correlation in the Multimodality Imaging Era

Radiological-pathological correlation is essential for diagnosing breast carcinoma and in assessing the subgross morphological prognostic parameters listed above. A pathologist who is not familiar with the radiological findings when processing a preoperative biopsy or an operative specimen is more likely to make mistakes. Testing the concordance between the radiological and histological findings is not a matter of just comparing the values provided by these methods. Deviating data may result from technical/natural factors. The breast is hanging during the magnetic resonance imaging examination and the antero-posterior axis of the breast becomes transiently longer than when the patient is in an upright position. During mammography, the breast is compressed to a certain level, and the cranio-caudal axis becomes shorter. The breast tissue is much softer than the tumor itself and is easily deformed when placed on the firm surface of a transport plate or the bottom of a formalin-filled dish. Formalin fixation will cause shrinkage of the specimen, but deformation of the specimen during fixation in a dish of inadequate size may cause much more obvious discrepancies. The most common cause of discrepancies is, however, failure in the radiological-pathological correlation. 

**Figure 5:**
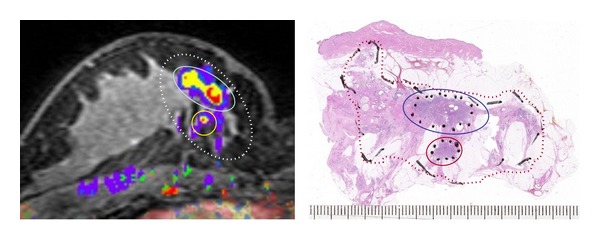
Magnetic resonance imaging-large-format histopathology correlation in a tumor with two invasive foci and a diffuse in situ component (combined pattern diffuse). Dotted lines indicate the extent of the disease, 57 × 30 mm. Tumor size (the largest dimension of the largest invasive focus) is 16 mm. The magnetic resonance image is courtesy of Dr. Mats Ingvarsson.

Modern multimodality breast radiology is very accurate in determining the subgross morphological prognostic parameters [7]. It uses different imaging modalities for the same lesion, which when combined can compensate for the limitations of the results of the individual methods. Tables 2 and 3 show our preliminary results regarding tumor size measurement with the imaging methods of mammography plus ultrasound versus magnetic resonance imaging as compared with the findings in large-format histological sections. As mentioned previously, it is not realistic to expect a perfect millimetric concordance of the radiological and the histological values; rather, the findings should be categorized in clinically important groups, like early versus more advanced breast cancer, or nonextensive versus extensive tumors. The concordance analysis only means comparing the results without naming a gold standard method; histopathology is as likely to underestimate or overestimate the subgross parameters as the radiological methods. Concordant results were reached in at least 80% of our cases when the cases were categorized by tumor size into early and more advanced categories (Table 2). Similar levels of concordance were reached when diagnosing extensive tumors. However, a substantial proportion of cases characterized radiologically as nonextensive turned out to actually be extensive in the histological examination (Table 3). These discrepant cases corresponded to radiologically occult, most often noncalcified, low-grade multifocal or diffuse in situ carcinomas (72/162 cases) or to radiologically occult, most often <5 mm in size, invasive tumor foci (78/162 cases). Very rarely, large diffuse invasive breast carcinomas were radiologically occult or manifested with nonspecific signs. The magnetic resonance imaging-large-format histopathology correlation of a case of breast carcinoma with multifocal invasive and diffuse in situ components is shown in Figure 5.

## 6. Conclusions

Most breast carcinomas exhibit both in situ and invasive components. Although up to 70% of invasive tumors have only an unifocal invasive component, most breast carcinomas have a complex morphology when the distribution of the in situ and invasive components are combined. This complexity is evident both at early and more advanced stages of the disease. Half of breast cancer cases are extensive and occupy a tissue volume measuring ≥40 mm in the greatest dimension. Tumor size, disease extent, and lesion distribution are essential parameters for planning appropriate therapy and also have very significant prognostic power. Proper assessment of these parameters requires additional effort from the pathologists, including a detailed and systematic radiological-pathological correlation in every case of breast cancer. The method of large-format histopathology is a prerequisite for such correlations.

## Figures and Tables

**Table 1:** 1000 consecutive breast cancer cases by focality, disease extent, and stage. Falun, Dec 2007 to Jun 2012.

	Unifocal % (*n*/*N*)		Multifocal % (*n*/*N*)		Diffuse % (*n*/*N*)		Total % (*n*/*N*)
	Extensive	Nonextensive	Extensive	Nonextensive	Extensive	Nonextensive
In situ	0	31 (44/144)	17 (25/144)	11 (17/144)	3 (44/144)	10 (14/144)	14 (144/1000)
Early Invasive	0	40 (140/349)	20 (68/349)	14 (48/349)	22 (78/349)	4 (15/349)	35 (349/1000)
Advanced	3 (16/500)	33 (166/500)	30 (148/500)	8 (41/500)	22 (109/500)	4 (20/500)	50 (500/1000)
Extent	4 (16/366)	96 (350/366)	69 (241/347)	31 (106/347)	83 (231/280)	17 (49/280)	99 (993/1000)

Total	36% (366/1000)		35 (347/1000)		28 (280/1000)		100* (1000/1000)

*Disease extent was undetermined in 7 cases.

**Table 2:** Concordance of radiological and pathological size categories in 647 consecutive breast cancer cases, Falun, 2008–2011.

Tumor size category	Large-format histopathology versus magnetic resonance imaging concordance % (*n*/*N*)	Large-format histopathology versus mammography + ultrasound concordance % (*n*/*N*)	Size distribution of the cases in the same period % (*n*/*N*)
Early invasive cancer (<15 mm)	79 (87/110)	74 (172/231)	39 (255/647)
More advanced (≥15 mm)	80 (213/264)	92 (254/276)	61 (392/647)
All histologically verified	80 (300/374)	84 (426/507)	100 (647/647)

**Table 3:** Concordance of radiological and pathological extent categories in 675 consecutive breast cancer cases, Falun, 2008–2011.

Radiological extent category	Large-format histopathology extent categories
Nonextensive (<40 mm) 72% (486/675)	Nonextensive 66% (321/486)
	Extensive 33% (162/486)
	3 cases not assessable

Extensive (≥40 mm) 28% (189/675)	Non-extensive 13% (24/189)
	Extensive 84% (159/189)
	6 cases not assessable

Overall concordance	71% (480/675)
